# Food handling practices and associated factors among food handlers working in public food and drink service establishments in Woldia town, Northeast Ethiopia

**DOI:** 10.11604/pamj.2021.40.128.19757

**Published:** 2021-11-02

**Authors:** Melese Abate Reta, Mekonnin Tesfa Lemma, Ashete Adere Gemeda, Getasew Assefa Lemlem

**Affiliations:** 1 Department of Medical Laboratory Science, College of Health Sciences, Woldia University, Woldia, Ethiopia,; 2 Department of Nursing, College of Health Sciences, Woldia University, Woldia, Ethiopia,; 3 Department of Midwifery, College of Health Sciences, Woldia University, Woldia, Ethiopia,; 4 Department of Statistics, Faculty of Natural and Computational Sciences, Woldia University, Woldia, Ethiopia

**Keywords:** Food handlers, food handling practices, determinants, catering establishments, Woldia

## Abstract

**Introduction:**

foodborne disease (FBD) is a major public health problem globally. Inadequate food workers' knowledge, attitude, and low level of food handling practices (FHPs) may all contribute to the possibility of FBD outbreaks in public food service establishments. This study aimed to assess FHPs and associated factors among food handlers working in public food and drink service establishments in Woldia town, Northeast Ethiopia.

**Methods:**

an institutional-based cross-sectional study was conducted from 01 to 29, January 2017. A total of 288 food handlers were recruited through a simple random selection method. A structured interviewer-administered questionnaire and observation checklists were used to collect the respondents' socio-demographic characteristics, knowledge status on FHPs, and food handling working practices data. Descriptive statistics, bivariate and multivariate logistic regression analysis were employed using SPSS version 20 software. Those variables with a p< 0.05 were considered statistically significant.

**Results:**

out of 288 participants, 91.7% were female, and 82.3% were single, while 69.8% were literate. One hundred eighty-four (63.9%) of them were under 15-25 years of age, with a median age of 23.3 years. The proportion of good FHP was (n=134, 46.5%) (95% CI: 41.00-52.4%). Advanced age (adjusted odds ratio (AOR) =12.01, 95% CI: 1.96-73.52), education (participants who attend grades 7-12 (AOR=2.33, 95% CI: 1.14-4.79), and above secondary education (AOR=2.29, 95% CI: 1.05-4.61), work experience above six years (AOR=2.43, 95% CI: 2.08-3.17), received formal training (AOR=1.79, 95% CI: 1.68-4.71), and inspection visits by a concerned body (AOR=2.24, 95% CI: 1.05-3.09) were factors positively associated with handling practices.

**Conclusion:**

the study revealed that FHP in the study area was low. Age, education, service year, training received and sanitary inspection visits by the regulatory personnel were factors significantly associated with FHPs. This finding highlights the importance of employing regular sanitary inspection visits to public food service establishments by the concerned authority to ensure that all food handlers have the knowledge and the skill to provide safe food.

## Introduction

Foodborne disease (FBD) is a major public health problem globally [1, 2]. In each year, an enormous number of individuals are at increased risk, and many of them get ill and die because of the consumption of contaminated foods [2]. The problem is more serious in developing countries due to food workers' inefficient food handling and sanitation practices, a lack of food safety standards, ineffective supervision systems, and food workers' low level of education, knowledge, and attitude [1-3]. Although the burden of FBD is a major public health concern globally, the World Health Organization (WHO) Regions, particularly Africa and South-East Asia Regions, have higher incidence and death rates, including among under 5 children [4]. In the WHO African Region, over 91 million individuals are estimated to fall ill and 137,000 dies every year because of FBDs. Diarrheal diseases contribute to 70% of FBDs in the region [4]. According to the WHO report, over 2.2 million people die each year as a result of food and water contamination, the majority of whom are children living in developing countries. Typhoid fever affects 16.6 million people worldwide and is responsible for 600,000 deaths each year [5].

Although the FBD is the main public health concern, ensuring food safety to protect people from different FBDs continues a substantial challenge, globally [6-8]. Several previous studies evidenced that the most contributing factors for potential FBD outbreaks in food service establishments are due to unsanitary conditions [9, 10], food handler's poor personal and food hygiene practices [2, 9-14], and the low level of food handling knowledge, and attitude [15-17]. When food is contaminated with certain pathogenic bacteria, viruses, or parasites, it can cause food-borne illnesses [18]. Consumption of unsafe food contributes to the transmission of over 200 known diseases [19]. Consequently, about two billion illnesses are associated with FBDs [2].

Among the most prevalent pathogens that cause food contamination and outbreaks as a result of unsanitary conditions in establishments, food handlers' lack of knowledge and attitude toward safe food handling, the following are a few species that have been mentioned in various studies: *Shigella spp* [13, 20-22], *Salmonella spp* [21-24], *Staphylococcus aureus* [22, 25-33], *Listeria monocytogenes* [34], *Escherichia coli* [12, 35, 36], *Giardia lamblia, Ascaris lumbricoid*, and *Entamoeba histolytica* [22, 24, 27], Norovirus [11, 37]. Since food-related diseases can be serious, or even fatal, it is important to know and practice safe food-handling behaviors to help reduce the risk of getting sick from contaminated food [18].

Food handlers can always contribute to ensuring food safety at a public food and drink service establishments [38]. A previous study had mentioned that 10-20% of FBDs are due to food contamination by food handlers [39]. A study confirmed by the Food and Drug Administration (FDA) determined that 81 FBDs were caused by foods contaminated via food processing workers [40]. Low hygiene of food handlers, inappropriate food cooking measures, and improper food utensil storage can contribute the way for pathogens to come into contact with food and cause FBDs in consumers [41]. Realizing the food handler's role in food contamination and disease outbreaks, the WHO developed guidelines to ensure that food workers receive proper training regarding their roles and responsibilities during food preparation and handling [8, 42]. Hence, realizing food safety measures and possible factors that can be the source of FBD is important for all food handlers in controlling and preventing FBDs [42, 43].

Several studies conducted in a variety of study contexts showed a variety of sociodemographic and other predictors related to FHPs. Of those, food safety training [3, 10, 44], the service year of food workers [3, 44], advanced age [44], educational status [3, 38], marital status [1, 44], monthly income [1, 44], and knowledge level [1, 10, 44, 45] were factors significantly associated with FHPs. Moreover, other environmental factors at the establishment, such as ineffective liquid and solid waste management systems, inadequate water supply, and the lack of handwashing, shower, and toilet facilities, were found to be associated with the FHPs [1].

In Ethiopia, there is scant information that reveals the magnitudes of FBDs due to improper food safety practices in food and drink service establishments. However, a few earlier studies conducted in various parts of the country found that public food service establishments have a high rate of unhygienic conditions [10, 46, 47]. Woldia town, the capital of North Wollo zone administration, and the focus of this study is urbanizing at a fast rate. It is a stop-over for tourists and the public at large travelling to and from Bahir Dar city, Lalibela, Mekelle, and Afar Region. Hence, many people make use of the food, drink, and accommodation services in the town. Hence, assessing and providing information regarding food workers' level of handling practices and determinants has a vital role in refining FHPs among food handlers working at food and drink services establishments in the study area. Therefore, this study aimed to assess the level of FHPs and associated factors among food handlers working at a public food and drink service establishment at Woldia tow, Northeast Ethiopia.

## Methods

**Figure 1:**
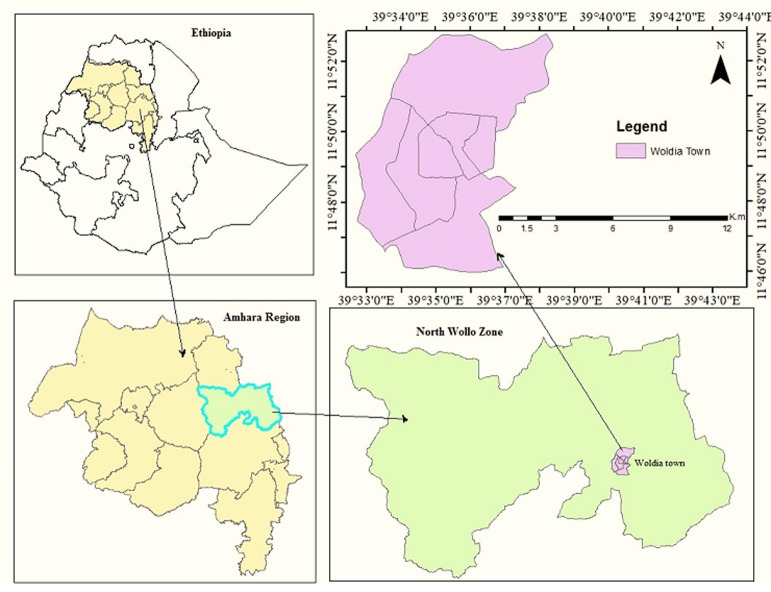
study area map

**Study setting and design:** an institutional-based cross-sectional study was conducted from 01^st^ to 29^th^ January 2017. Woldia town is the capital of the North Wollo zone administration and is found in the Amhara Regional State, Northeast of Ethiopia at 11°49'59.99" N latitude and 39°40'59.99"E longitude, at 2,112 meters above sea level (Figure 1). The town is 370km away from the regional capital, Bahir Dar, and 521km from the Ethiopian capital, Addis Ababa [47]. All food workers and handlers working at public food and drink service establishments in the town were the focus of this study. For our proper sampling techniques, a total list of public food and drink service establishments and the total number of food handlers were found from Woldia town Trade and Industry office. As it is depicted in [47], a total of 408 public food and drink service establishments were legally registered by the town Trade and Industry office during data collection. At those establishments, a total of 956 (302 males and 654 females) food handlers were working at the time of data collection.

**Study population:** all food workers and handlers working at the public food and drink establishments in the town were the focus of this study. As mentioned above, a list of 956 (302 males and 654 females) food handlers were found from the town's Trade and Industry office and used as the source of the study population.

**Sample size and sampling techniques:** the sample size was calculated using a single population proportion formula, considering 95% CI, marginal error of 5%, the proportion (52.5%) [1], and 5% non-response rate. Accordingly, the final sample size was calculated and corrected to be 288. As mentioned above, for proper sampling technique, lists of the establishments (n=408), and food handlers (n=956) were found from the town Trade and Industry office [47]. Then, lists and the number of food handlers who were working at each establishment during data collection were obtained from each establishment owner/manager. Due to the presence of more than one food handler in each establishment, one food handler per establishment was selected using a simple random sampling method.

**Data collection:** a structured interviewer-administered questionnaire and an observational checklist were used to collect the respondents' socio-demographic characteristics, knowledge status on proper FHPs, and food handling working practices data. All data collection processes were performed during the establishment's working time; moreover, the food handlers were not aware of the researchers` visit for the data collection. The questionnaire was developed through reviewing previously published literatures [1, 16, 38, 48], and adopted from the WHO [8]. The developed questionnaire was subjected to a preliminary validation [48], peer-reviewed, and pilot tested to assess its clarity, the suitability of wording, and the average time needed for its completion. Based on the pilot study, necessary modifications were identified and resolved before a final version was administered, whereas its results were not included in the final analysis.

The questionnaire was structured into three distinctive sections. Section one was to collect information about food handlers' socio-demographic characteristics such as sex, age, marital status, level of education, religion, monthly income (in birr), and length of employment in public food and drink service establishments. Section two was concerned with the food handlers' knowledge status on proper FHPs. This section of the questionnaire dealing with food hygienic knowledge comprised eight close-ended questions with multiple possible answers. These questions specifically dealt with food handlers' knowledge of personal hygiene, food contamination, about FBDs, mode of transmission for FBDs, temperature control, and hygienic practices. A scale ranging between 0 and 8 (representing the total number of questions on food handling knowledge) was used to evaluate the overall knowledge of respondents. Food-handlers that obtained a total score less than or equal to the mean value were considered having a “low” knowledge level and those that had a score greater than the mean value (>50% accuracy) were considered having “good/ or adequate” knowledge.

In section three, which dealt with FHPs, the good handling practices of food handlers were assessed and evaluated based on observational checklists (additional file: Annex 1). The section had eighteen questions/statements. Each correct practice reported scored one point. For evaluation, a score >50% by an individual was considered as having “good/ or adequate” FHPs. Visual observations of the FHPs and food handlers' knowledge on proper FHPs were carried out by three trained BSc nurses who have experience in similar data collection, one food safety and environmental sanitation inspection expert under the supervision of one certified sanitation inspector. The quality of collected data was assured through pilot-testing, translating the tool to the local language (Amharic), and translated back to the English language to check its consistency, training to the data collectors, and lastly collected data was checked for its completeness and cleaned for analysis.

### Operational definitions

**Food handlers:** anyone who works at a public food and drink service establishment and who handles food or who has direct contact with any food utensils, such as cutlery, dishes, plates, or chopping boards [1, 49].

**Ethical consideration:** the study was ethically reviewed and approved by Woldia University research ethics review committee, and permission was also found from the Municipality Office and the district health department of Woldia town. Data were collected after written consent was obtained from the public food and drink service establishment owners and interviews follow the full consent of the food handlers. Participants were assured that all information they provided was kept confidential and used only for the aim of this study.

**Data analysis:** the results were presented using descriptive statistics. The binary logistic regression model was used to identify associated factors with the outcome variable. The odds ratio with 95% CI was calculated to show the strength of associations. Variables with a p<0.05 in the bivariate analysis were entered into the multivariate analysis. In the multivariate analysis, variables with a p<0.05 were considered statistically significant.

## Results

**Socio-demographic characteristics:** Table 1 illustrates the summarized demographic profile of respondents in this study. A total of 288 food handlers have participated, and the median age was 23.3years. Of the total, 264 (91.7%) were females, while 237 (82.3%) were single. The majority (n=201, 69.8%) were literate who attained education up to grade twelve and above. Nevertheless, fewer (n=42, 14.6%) participants had taken formal training on proper food preparations and handling practices, of those, (n=7, 16.7%) had received the certificate. More than half (n=173, 60.1%) of food handlers had 1-5 years of work experience (Table 1).

**Table 1:** socio-demographic profiles of food handlers working in public food and drink service establishments in Woldia town, Northeast Ethiopia, 2017

Characteristics		Frequency (n)	Percent (%)
Sex	Male	24	8.3
	Female	264	91.7
Age (year)	15-20	94	32.6
	21-25	90	31.3
	26-30	78	27.1
	31-35	17	5.9
	36-40	9	3.1
Marital status	Single	237	82.3
	Married	49	17
	Divorced	2	0.7
Educational attainment	Illiterate	87	30.2
	Grade 1-6	98	34
	Grade 7-12	90	31.3
	> Grade 12	13	4.5
The religion of a food handler	Orthodox	255	88.5
	Muslim	18	6.3
	Protestant	10	3.5
	Catholic	5	1.7
Food handler's monthly income (in birr)	≥500	186	64.6
	<500	102	35.4
Food handling and hygiene training received	Yes	42	14.6
	No	246	85.4
If food handlers received training, does he/she certify? (n=42)	Yes	7	16.7
	No	35	83.3
The service year of food handlers	<1 year	98	34.0
	1-5 year	173	60.1
	6-10 year	11	3.8
	>10 years	6	2.1

**Food handler's knowledge on FHPs:** to assess the knowledge status of FHPs, participants were asked eight knowledge-based questions. Of the total, (n=182, 63.2%) had adequate knowledge status on FHPs. More than half (n=179, 62.2%) of them have heard about FBDs, of which 61.1% had good knowledge status. About half (n=145, 50.3%) of food handlers had reported that the causes of FBDs are germs, while (n=110, 38.2%) had mentioned the cause as an unapproved source. The majority (n=109, 37.8%) of food handlers did not know the mode of FBD transmission. Regarding the reasons mentioned for food contamination, (n=106, 36.8%) claimed a dirty utensil as a cause, whereas 37.5% mentioned an unapproved source. Of the total, (n=205, 71.2%) of them mentioned that good personal hygiene can prevent FBDs. The majority (n=190, 66%), and (n=191, 66.3%) had realized that raw milk and raw meat can transmit different diseases, respectively (Table 2).

**Table 2:** knowledge status of food handlers on FHPs working in public food and drink service establishments in Woldia town, Northeast Ethiopia, 2017

Characteristics	Categories	Total, n (%)	Knowledge status score	
			Good, n (%)	Poor, n (%)
Do you hear about FBDs?	Yes	179 (62.2)	176 (61.1)	3 (1.0)
	No	109 (37.8)	6 (2.1)	103 (35.8)
Causes of FBDs	Germs	145 (50.3)	145 (50.3)	0 (0.0)
	Adding chemicals	6 (2.1)	6 (2.1)	0 (0.0)
	Anger of the God	1 (0.3)	1 (0.3)	0 (0.0)
	Unapproved sources	110 (38.2)	7 (2.4)	103 (35.8)
	Don't know	26 (9.0)	23 (8)	3 (1.0)
What is the mode of transmission of FBDs?	Contaminated food	104 (36.1)	103 (35.8)	1 (0.3)
	Contaminated water	65 (22.6)	63 (21.9)	2 (0.7)
	Infected food handlers	10 (3.5)	10 (3.5)	0 (0.0)
	Don't know	109 (37.8)	6 (2.1)	103 (35.8)
The reasons for food contaminations	Dirt hands	24 (8.3)	24 (8.3)	0 (0.0)
	Infected food handlers	33 (11.5)	32 (11.1)	1 (0.3)
	Dirt utensil	106 (36.8)	103 (35.8)	3 (1.0)
	Dirt working environment	6 (2.1)	6 (2.1)	0 (0.0)
	Unapproved sources	108 (37.5)	6 (2.1)	102 (35.4)
	Don't know	11 (3.8)	11 (3.8)	0 (0.0)
The danger temperature zone for potentially hazardous foods	Below 5oC	58 (20.1)	1 (0.1)	57 (19.8)
	5-60oC	189 (65.6)	180 (62.5)	9 (3.1)
	Above 60oC	22 (7.6)	1 (0.3)	21 (7.3)
	Don't know	19 (6.6)	0 (0.0)	19 (6.6)
Can raw milk transmit diseases?	Yes	190 (66)	173 (60.1)	17 (5.9)
	No	72 (25)	6 (2.1)	66 (22.9)
	Don't know	26 (9.0)	3 (1.0)	23 (8.0)
Can raw meats transmit disease?	Yes	191 (66.3)	173 (60.1)	18 (6.3)
	No	70 (24.3)	6 (2.1)	64 (22.2)
	Don't know	27 (9.4)	3 (1.0)	24 (8.3)
Does good personal hygiene prevent from FBDs?	Yes	205 (71.2)	162 (56.3)	43 (14.9)
	No	64 (22.2)	15 (5.2)	49 (17.0)
	Don't know	19 (6.6)	5 (1.7)	14 (4.9)

**Food handling practices:** this study revealed that the level of good FHP was 46.5% (95% CI: 37.01-56.2%). The observational assessment had shown that over three-fourths (n=222, 77.1%) of participants did not wear outer garments/gowns, of which 44.4% had low FHPs scores. The majority (n=253, 87.8%) of food handlers had not covered their hair during food handling and preparation, while (n=174, 60.4%) participants' fingernails had not short trimmed and clean. Moreover, (n=140, 48.6%) had worn any type of jewellery on their hand at the time of data collection, while (n=128, 44.4%) participants had not used soap/or detergent for washing dishes. Over three-fourth (n=229, 79.5%), and (n=267, 92.7%) of participants had used soap and wash their hands before working with foods and after visiting a latrine, respectively. Furthermore, 72.2% and 82.6% of participants had not preserved ready-to-eat foods in a hygienic container and had not carefully kept food utensils on the shelf, respectively. More than half (n=154, 53.5) had not washed their utensils using three washing compartments, and they had inadequate FHPs. The majority (n=245, 85.1%) had not taken medical checkups in the past six months, while 52.4% were not inspected by concerned authorities for the last six months (Table 3).

**Table 3:** observational assessment results on food handlers' FHPs in public food and drink service establishments in Woldia town, Northeast Ethiopia, 2017

Characteristics		Total, n (%)	FHP score	
			Good, n (%)	Poor, n (%)
Do food handlers wear outer garments/gowns during the visit?	Yes	66 (22.9)	40 (13.9)	26 (9.0)
	No	222 (77.1)	94 (32.6)	128 (44.4)
If they wear outer garments/gowns, do the garments/gowns were clean? (n =66 )	Yes	11 (16.7)	9 (13.6)	2 (3.0)
	No	55 (83.3)	33 (50)	22 (33.3)
Do food handlers cover their hair while working?	Yes	35 (12.2)	27 (9.4)	8 (2.8)
	No	253 (87.8)	107 (37.2)	146 (50.7)
Do food handlers' fingernails short trimmed and clean?	Yes	114 (39.6)	70 (24.3)	44 (15.3)
	No	174 (60.4)	64 (22.2)	110 (38.2)
Do food handlers wear any type of jewelry/ring on their hands at the time of the visit?	Yes	140 (48.6)	46 (16.0)	94 (32.6)
	No	148 (51.4)	88 (30.6)	60 (20.8)
Clean the work surfaces after each task	Yes	218 (75.7)	116 (40.3)	102 (35.4)
	No	70 (24.3)	18 (6.3)	52 (18.1)
Used soap/detergent for washing dishes	Yes	160 (55.6)	133 (46.2)	27 (9.4)
	No	128 (44.4)	1 (0.3)	127 (44.1)
Used hot water for washing dishes	Yes	138 (47.9)	134 (46.5)	4 (1.4)
	No	150 (52.1)	0 (0)	150 (52.1)
Wash their utensils using three washing compartments	Yes	134 (46.5)	134 (46.5)	0 (0)
	No	154 (53.5)	0 (0)	154 (53.5)
Did food handlers wash the chopping board and knife with soap or belch after using?	Yes	142 (49.3)	134 (46.5)	8 (2.8)
	No	146 (50.7)	0 (0)	146 (50.7)
Did food handlers wash their hands with detergent and water before working with food?	Yes	229 (79.5)	107 (37.2)	122 (42.4)
	No	59 (20.5)	27 (9.4)	32 (11.1)
Did food handlers wash their hands with detergent and water after visiting the toilet?	Yes	267 (92.7)	129 (44.8)	138 (47.9)
	No	21 (7.3)	5 (1.7)	16 (5.6)
Did food handlers keep ready-to-eat foods in a hygienic container?	Yes	80 (27.8)	75 (26.0)	5 (1.7)
	No	208 (72.2)	59 (20.5)	149 (51.7)
Did food handlers carefully keep food utensils on the shelf/or cabinet?	Yes	50 (17.4)	50 (17.4)	0 (0)
	No	238 (82.6)	84 (29.2)	154 (53.5)
Did food handlers keep uncooked foods separate from cooked food?	Yes	203 (70.5)	116 (40.3)	87 (30.2)
	No	85 (29.5)	18 (6.3)	67 (23.3)
Did food handlers store perishable ready-to-eat foods in the refrigerator?	Yes	191 (66.3)	110 (38.2)	81 (28.1)
	No	97 (33.7)	24 (8.3)	73 (25.3)
Did food handlers take a medical checkup in the past six months?	Yes	43 (14.9)	35 (12.2)	8 (2.8)
	No	245 (85.1)	99 (34.4)	146 (50.7)
Does the establishment and food handlers were inspected by regulatory personnel in the past six months?	Yes	137 (47.6)	86 (28.9)	51 (17.7)
	No	151 (52.4)	48 (16.7)	103 (35.8)

**Factors associated with FHPs:** in the multivariate logistic regression analysis; age, education, service years, training received and sanitary inspection visits by experts from the town sanitation office were factors associated with FHPs (p<0.05). The results revealed that participants under 36-40 years of age have higher odds of adequate FHPs as compared to participants under 15-20 years of age (AOR=12.01, 95% CI: 1.96-73.52). Compared to illiterate participants, the odds of having adequate FHPs were higher in participants who had attended grades 7-12 (AOR=2.33, 95% CI: 1.14-4.79) and above secondary education (AOR=2.29, 95% CI: 1.05-4.61). A statistically significant association between service years of participants and adequate FHPs was found in this study. Thus, the odds of having adequate FHP was higher among participants with longer service years' experience (6-10years) (AOR=2.43, 95% CI: 2.08-3.17). Food handlers who had received formal training on food preparation and handling practices had higher odds of adequate FHPs than those who didn't (AOR=1.79, 95% CI: 1.68-4.71). The regulatory body's frequent inspection visit to the catering establishments is significant to encourage and assure good sanitation practices. In our findings, food handlers who had been inspected by concerned authorities for the past six months had higher odds of good FHPs as compared to those who had not inspected (AOR=2.24, 95% CI: 1.05-3.09) (Table 4).

**Table 4:** factors associated with FHPs among food handlers working in public food and drink service establishments in Woldia town, Northeast Ethiopia, 2017

Characteristics		FHP score		COR (95% CI)	p-value	AOR (95% CI)	p-value
		Good	Poor				
Sex	Male	17	7	1		1	0.091
	Female	117	147	3.05(1.22-7.60)	0.04	2.93(1.81-10.68)	
Age (years)	15-20	58	36	1		1	
	21-25	42	48	1.84(1.02-3.31)	0.041	2.09(1.05-4.17)	0.067
	26-30	25	53	3.41(1.82-6.42)	0.000	4.51(1.79-11.33)	0.031
	31-35	6	11	2.95(1.01-8.68)	0.009	10.69(2.04-56.04)	0.005
	36-40	3	6	3.22(1.76-13.70)	0.013	12.01(1.96-73.52)	0.017
Marital status	Single	107	130	1		1	
	Married	27	22	2.95(1.01-8.68)	0.025	2.09(1.05-4.17)	0.075
	Divorced	0	2	-	-	-	-
Educational attainment	Illiterate	19	68	1		1	
	Grade 1-6	40	58	2.41(1.21-3.78)	0.016	2.91(1.41-4.99)	0.057
	Grade 7-12	65	25	1.11(1.05-2.21)	0.001	2.33(1.14-4.79)	0.019
	Grade >12	10	3	2.08(1.02-5.34)	0.0001	2.29(1.05-4.61)	0.016
Food handler's monthly income (in birr)	≥ 500	112	74	3.18(2.10-4.32)	0.006	2.29(1.14-5.61)	0.091
	< 500	22	80	1		1	
The service year of food handlers	<1 year	46	52	1		1	
	1-5 year	74	99	1.18(0.72-2.95)	0.507	0.55(0.29-1.07)	0.077
	6-10 year	8	3	1.33(1.08-2.33)	0.018	2.43(2.08-3.17)	0.034
	> 10 years	6	0	-	-	-	-
Food handling and preparation training received	Yes	22	20	1.76(1.40-3.46)	0.012	1.79(1.68-4.71)	0.037
	No	112	134	1		1	
The sanitary inspection visits	Yes	86	51	3.28(1.17-6.45)	0.000	2.24(1.05-3.09)	0.028
	No	48	103	1		1	

**Food handling practices versus sanitary inspection visits:** during the time of observational assessment, food establishments and food workers were assessed whether the establishment's environmental sanitary conditions were inspected by the concerned authority for the last six months or not. Of the total, only 137 (47.6%) (95% CI: 42.00-53.50%) of catering establishments and food workers were inspected by concerned authorities in the past six months. In the multivariate-adjusted model, we found a significant interaction between the regulatory body's frequent inspection visits and adequate FHPs (p<0.05). Food handlers who were working in the food and drink service institutions that were inspected by concerned authorities in the past six months had higher odds of proper storage of food utensils (AOR=2.95, 95% CI: 1.28-4.23), had trimmed fingernails (AOR=3.38, 95% CI: 1.18-7.812) and had washed their hands with soap after the toilet (AOR=2.21, 95% CI: 1.03- 4.27) as compared with those who were worked in establishments that hadn't been inspected (Table 5).

**Table 5:** FHPs versus sanitary inspection visits by regulatory personnel in Woldia town, Northeast Ethiopia, 2017

Characteristics		Sanitary Inspection		COR (95% CI)	p-value	AOR (95% CI)	p-value
		Yes	No				
Storage of food utensils	Proper	30	20	1.55 (0.29-2.01)	0.005	2.95 (1.28-4.23)	0.008
	Improper	107	131	1		1	
Food handlers with trimmed fingernails	Yes	72	42	2.35 (1.21-4.57)	0.001	3.38 (1.18-7.812)	0.013
	No	65	109	1		1	
Food handler's hair was covered	Yes	28	7	4.19(1.08-10.49)	0.015	3.32 (1.11-7.94)	0.079
	No	109	144	1		1	
Food handler washes his/her hands before starting the food handling	**Yes**	114	115	1.64 (0.36-3.16)	0.034	2.53 (1.24-4.16)	0.072
	No	23	36	1		1	
Washing hands after visiting the toilet with soap and water	Yes	134	133	3.17 (1.48-9.58)	0.001	2.21 (1.03-4.27)	0.040
	No	3	18	1		1	
Washing utensils using three compartments	Yes	86	48	3.28 (1.17-7.45)	0.048	3.20 (1.01-12.50)	0.080
	No	51	103	1		1	
Using soap/detergent for washing dishes	Yes	90	70	3.45 (1.28-7.73)	0.004	2.23 (1.65-7.70)	0.095
	No	47	81	1		1	
Stored perishable ready- to- eat foods in the refrigerator (n=226)	Yes	53	7	2.17 (0.17-4.47)	0.002	2.20 (1.08-7.49)	0.061
	No	70	96	1		1	

## Discussion

Improper FHP is one of the major ways for FBDs transmission. An emphasis needs to be given to FHPs by the concerned authorities. Food handlers often have little understanding of the risks of microbial or chemical contamination of food or how to avoid them [8]. Since food-related diseases can be serious, or even fatal, it is important to know and practice safe food handling behaviors to help reduce the risk of getting sick from contaminated food [18]. Therefore, this study provides an insight into the status of FHPs in the study area. Our study revealed that the level of good/or adequate FHP was 46.5% (95% CI: 37.01-56.20). This result is lower than those found in previous studies conducted in different places in Ethiopia: Dangla at 52.2% [1], Bahir Dar 67.6% [3], Mekelle 63.9% [45], and Dire Dawa 52.4% [50]. Similarly, this finding is lower than other studies conducted in Malaysia at 59.30% [51], Jordan at 89.43% [52], and Nigeria at 54.7% [53]. The difference might come across due to study settings, differences in participants' sociodemographic characteristics. For instance, the study in Malaysia was conducted on a university campus, whereas in Jordan the study was conducted in a hospital setting. In fact, those institutions are assumed to have adequate resources and a suitable setup for FHPs as compared to our study area. Besides this, the education level of food handlers in Malaysia and Jordan might contribute to the variation. The proportion of food handlers who attended secondary school and above were 77% and 94% in Malaysia and Jordan studies, respectively, whereas in our study only 35.8%. As education level progresses, food handlers would have improved knowledge and attitude towards good FHPs [54].

However, this finding is higher than the report from Nigeria 36.5% [55], Gondar, Ethiopia 30.3% [56], Gamogofa, Ethiopia 32.6% [57], and Debark, Ethiopia 40.1% [58]. This might be due to the difference in the study period and the cut-off points used to calculate the level of FHPs. The study conducted in Gondar town got around eight years long. Due to globalization, access to information improved from time to time, and food handlers can develop a good knowledge and positive attitude towards food handling so that they could perform good FHPs relatively better [59].

In addition to this, the cut-off points used to calculate the level of FHPs were determined in three levels (good, fair, and poor) [50, 60] as compared to this study in which the level of FHP was determined into two levels using the minimum cut-off point (good and poor). The cut-off point difference entirely changes the results of the study. In the study conducted at Gamogofa, interviewees were mostly males and had a primary school and below 68.66% as compared to this study in which only 8.3% of males and 64.2% of primary school and below education level of respondents involved. This is because females had more experience even in their day-to-day home activity than males and the low education level of food handlers will have poor knowledge and attitude so that not liable to apply basic good handling principles [50]. In this study, the odds of performing good FHPs among food handlers who were of advanced age (31-36 years) were 10.7 times higher as compared to those who were younger (15-20 years). This result is consistent with previous study reports [44, 61]. The possible reason for this could be the fact that proper FHPs behavior can be improved when their age increased and develop the experience to handle food safely [44, 61].

In this study, as compared to illiterates, performing good FHP was 2.33 and 2.29 times higher among those who had attained grades 7-12 and above grade 12, respectively. This finding was consistent with the study conducted in Bahir Dar [3], Dire Dawa [50], Addis Ababa [54], Italy [62], Jordan [52], Ghana [48], and Nigeria [63]. This is because the depth of knowledge could affect the FHPs and education can help to enhance knowledge, thereby developing the skills of food handlers to work according to the standard procedures to maintain food safety [38, 52, 54, 64].

Besides, food handlers who had 6-10 years of work experience were 2.43 times likely to have good FHPs as compared to their counterparts. This finding was consistent with the study conducted in Bahir Dar [3]. Similarly, Gizaw *et al*. (2014) had mentioned that food handlers who had 3years of experience were 3.37 times likely to have good FHPs [44]. Proper FHP status may raise as the food handler's service year increases [61]. This might be the reason that good FHP behavior can be acquired by continuous practices; hence, food handlers who had such experience are in a good position to enhance skills in FHPs [44, 61].

Food handlers who had received formal training were 1.79 times likely to have good FHPs as compared to those who hadn't. Few earlier studies supported this finding [3, 52, 65]. This might be training on FHPs can improve food handlers' knowledge about FBDs and related food safety issues [42, 65], and this enables them to have a better understanding and realize their responsibilities and FHP skills [65]. Food handling staff should receive instruction in proper FHPs and personal hygiene and should be required to undergo a test of their knowledge of the subject, and refresher courses should be given periodically throughout employment [8].

Furthermore, food handlers who were working in establishments inspected by concerned regulatory bodies in the past six months had performed proper storage of food utensils, had trimmed fingernails, washed their hands after visiting the toilet. These results were supported by an earlier study, which revealed that those establishments that had been supervised by regulatory bodies were more likely to fulfill the requirements of acceptable hygienic and sanitary practices [47]. The possible reason for this result might be regular sanitary supervision visits of the establishments supported by education can improve and sustain food handlers' proper FHPs and sanitary situations of the establishments.

## Conclusion

The relatively low level of FHPs in terms of washing and storage of food utensils, preserving ready-to-eat food in a hygienic container, wearing outer garments/or gowns, wearing any jewellery on their hand, and having untrimmed fingernails observed by data collectors at those establishments could contribute to higher consumption of risky food items and thus, higher susceptibility to FBDs. Moreover, this study revealed a significant association between participants' socio-demographic profile and their FHPs. Thorough and continuous training to food handlers and food establishment owners, regular sanitary inspection visits by concerned authority is highly recommended promoting proper sanitation facilities in those establishments and to ensure that all food handlers have the adequate knowledge and the skill to provide safe food. It is recommended that researchers, educators, food safety communicators, and the media should work towards educating the catering establishments, food handling staff, and the population to advance their proper food handling knowledge to safer food practices.

### What is known about this topic

- Although the FBD is the main public health concern, ensuring food safety to protect people from different FBDs continues a substantial challenge globally;
- Many food handlers' socio-demographic factors such as the service year, advanced age, educational status, marital status, monthly income, knowledge about FHPs were significantly associated with the FHPs of food handlers;
- Moreover, environmental conditions at public food service establishments such as inadequate liquid and solid waste management, water supply, handwashing, showering, and a toilet facility were shown to be associated with food handlers' FHPs.

### What this study adds

- The food handling training given to food handlers had enabled them to have adequate food safety knowledge, and proper FHPs and to keep their hygiene;
- The sanitary inspection visits by concerned authorities at least once in the past six months had a higher positive impact on food handlers to perform good FHPs;
- Moreover, food handlers who had worked in catering establishments that had been inspected by a concerned authority at least once in the past six months had good FHPs than those who hadn't.

## Annex

**Annex 1**: observational assessment results on food handler's FHPs in public food and drink service establishments in Woldia town, Northeast Ethiopia, 2017
